# Exploring Influence of Production Area and Harvest Time on Specialized Metabolite Content of *Glycyrrhiza glabra* Leaves and Evaluation of Antioxidant and Anti-Aging Properties

**DOI:** 10.3390/antiox13010093

**Published:** 2024-01-12

**Authors:** Teresa Docimo, Rita Celano, Alessia Lambiase, Rosa Di Sanzo, Simona Serio, Valentina Santoro, Paola Coccetti, Mariateresa Russo, Luca Rastrelli, Anna Lisa Piccinelli

**Affiliations:** 1 Institute of Bioscience and BioResources, National Research Council, 80055 Portici, Italy; teresa.docimo@ibbr.cnr.it; 2 Department of Pharmacy, University of Salerno, Via Giovanni Paolo II 132, 84084 Fisciano, Italy; sserio@unisa.it (S.S.); vsantoro@unisa.it (V.S.); rastrelli@unisa.it (L.R.); apiccinelli@unisa.it (A.L.P.); 3 National Biodiversity Future Center (NBFC), 90133 Palermo, Italy; a.lambiase1@campus.unimib.it (A.L.); paola.coccetti@unimib.it (P.C.); 4 Department of Biotechnology and Biosciences, University of Milano-Bicocca, 20126 Milano, Italy; 5 Department of Agriculture Science, Food Chemistry, Safety and Sensoromic Laboratory (FoCuSS Lab), University of Reggio Calabria, Via Salita Melissari, 89124 Reggio Calabria, Italy; rosa.disanzo@unirc.it (R.D.S.); mariateresa.russo@unirc.it (M.R.); 6 PhD Program in Drug Discovery and Development, University of Salerno, Via Giovanni Paolo II 132, 84084 Fisciano, Italy

**Keywords:** *Glycyrrhiza glabra* leaves, untapped resource, prenylated flavanone, prenylated dihydrostilbene, sustainable agriculture, bioeconomy, *Saccharomyces cerevisiae*, human α-synuclein, neurodegenerative disorders

## Abstract

Calabrian *Glycyrrhiza glabra* is one of the most appreciated licorice varieties worldwide, and its leaves are emerging as a valuable source of bioactive compounds. Nevertheless, this biomass is usually discarded, and its valorization could contribute to boost the economic value of the licorice production chain. In this study, the effects of production area and harvest time on the specialized metabolite content of *G. glabra* leaves (GGL) and also the antioxidant and anti-aging properties are evaluated to explore the potential of this untapped resource and to select the most optimal harvesting practices. GGL exhibited high levels of specialized metabolites (4–30 g/100 g of dry leaf) and the most abundant ones are pinocembrin, prenylated flavanones (licoflavanone and glabranin), and prenylated dihydrostilbenes. Their levels and antioxidant capacity in extracts are influenced by both production area and harvest time, showing a decisive role on specialized metabolites accumulation. Interestingly, GGL extracts strongly attenuate the toxicity of α-synuclein, the intracellular reactive oxygen species (ROS) content, and cellular senescence on *Saccharomyces cerevisiae* expressing human α-synuclein model, showing great potential to prevent aging and age-related disorders. These results provide insights into the phytochemical dynamics of GGL, identifying the best harvesting site and period to obtain bioactive-rich sources with potential uses in the food, nutraceutical, and pharmaceutical sectors.

## 1. Introduction

*Glycyrrhiza* species are flowering, herbaceous, perennial plants of the bean family Fabaceae, cultivated worldwide but native to Europe, Asia, and most of North America. The genus *Glycyrrhiza* comprises approximately thirty species, among which the European *G. glabra*, the Chinese *G. uralensis* and *G. inflata*, and the American *G. lepidota* are the most studied [1]. Roots and rhizomes of *G. glabra* (licorice) stand out for their well-documented and widely appreciated distinct characteristics related to botanical, culinary, and pharmacological properties [2]. Notably, licorice has a long history of therapeutic applications, including expectorant, carminative, anti-inflammatory, antiulcerous, antibacterial, antifungal, antiviral, antiallergic, and immunostimulant activities. Besides, it is also used as natural sweetener and flavor additive in foods, beverages, functional foods, and food supplements [2,3]. However, excessive consumption of licorice can lead to some side effects such as hypertension and edema [4]. A particularly distinctive feature of Calabrian *G. glabra* is its lower content of glycyrrhizin [5], which is the compound responsible for the sweet taste of roots but also for the hypertensive effects. The amount of this compound tends to vary depending on the root harvesting period, but also on factors such as soil conditions, climate, and agricultural practice. Indeed, the unique climatic and environmental conditions of the Calabrian region in Southern Italy have been shown having a deep impact on the distinctive aroma and sweetness of Calabrian licorice [5], which holds the “Protected Designation of Origin” (PDO) status.

Besides roots, this perennial plant has a vigorous vegetative biomass, which is emerging as a valuable source of bioactive compounds positively affecting human well-being. Nevertheless, during the blossom season, this biomass is an untapped resource, and its valorization could contribute indirectly to boost the economic value of licorice cultivation. Current harvesting practices of PDO licorice occur during the fall foliage season, when any leaves present are often discarded or left to decompose on the soil, resulting in poor quality and quantity of this biomass. Until now, root harvesting has prioritized yield over chemical composition, and a limited exploration into the influence of harvest times and environmental factors on the chemical and biological activity of root extracts has been reported. A recent study highlighted the impact of different harvesting periods on the chemical profile and bioactivity of *G. glabra* roots, envisaging alternative harvest times [6,7,8,9]. Regarding leaves, no studies have been conducted to investigate the effects of growth stage and environment on the phytochemical content and biological activities of *G. glabra* leaves. Knowledge of the influence of these factors on metabolic leaf content and the related potentiality might be crucial for envisaging either the most optimal harvesting practices for whole plant valorization or exploration of the potential uses of this untapped resource. These aspects could strongly improve the economic value of the Calabrian licorice production chain. Indeed, various untapped plant materials are currently being investigated as potential sources of bioactive compounds for the production of nutraceuticals and functional ingredients of food. Recent studies indicate that leaves of well-known crops and wild growing plants are unconventional and valuable source of functional and bioactive compounds [10,11].

*Glycyrrhiza* leaf extracts have been assayed for various biological activities, including antiproliferative, antimicrobial, antioxidant, anti-inflammatory, and α-glucosidase-inhibiting activities [12,13,14,15,16]. Recently, our UHPLC-HRMS profiling of aboveground and belowground tissues of Calabrian *G. glabra* revealed a different distinctive chemical composition of the leaves compared to the roots [17]. In agreement with [14], flavanones were found to be the most abundant class of secondary metabolites, with pinocembrin, licoflavanone, and glabranin as major compounds. The second distinctive class of phytochemicals of *G. glabra* leaves is represented by prenylated dihydrostilbenes, which together with prenylated flavanones represent distinctive chemical markers of leaves [17]. Among the chemical modifications of specialized metabolites, prenylation has the greatest impact on structural diversity and on biological activity. Compared with their non-prenylated counterparts, prenylation confers higher lipid solubility, higher capability to penetrate the membranes, and higher potential to interact with different cellular targets [18]. For this reason, prenylated phenolic compounds are promising molecules as drugs for their wide pharmacological spectrum of activities [19]. As an example, many plants within medicinal and food resources—such as *Morus alba*, *Humulus lupulus*, *Glycine max*, *Ficus carica,* and *Cannabis sativa*—accumulate prenylated phenols, and their extracts have been exploited in oncotherapy [20], metabolic disorders [21], and cardiovascular activities [22]. Regarding *Glycyrrhiza* metabolites, prenylated dihydrostilbenes and flavonoids from *G. uralensis* leaf extracts were investigated as therapeutic agents in liver fibrosis [12]. Recently, the potential of prenylated phenolics from Mulberry leaves has been also exploited for neuroprotective activity [20]. In the field of neurodegenerative diseases, accounting for a large and *increasing* health burden worldwide, drug discovery and development are urgently needed.

Neurodegenerative diseases, characterized by aberrant aggregates of the presynaptic protein α-synuclein, are collectively referred to as synucleinopathies, the second most common group of neurodegenerative diseases [23]. Despite the advances in the study of these pathologies, the detailed molecular mechanism of neuronal degeneration is still largely unknown. Several studies underline the relevant role of cellular models for a better understanding of the molecular regulation of synucleinopathies [24]. Many different models using yeast strains overexpressing human genes associated with diseases like Alzheimer’s disease (AD), Parkinson’s disease (PD), or Huntington’s disease (HD) have also been exploited in the last decades. Among them, one of the most studied yeast models concerns the expression of α-synuclein (α-syn) [25,26,27], a presynaptic protein whose alteration is associated with PD [23,28,29].

As *G. glabra* leaves (GGL) are a valuable source of beneficial compounds, to harness their potential, in this study we investigated the effect of the geographical area of cultivation and harvesting period on the profile of GGL specialized metabolites and their biological activities. To do so, a quantitative profiling of the main GGL compounds from *G. glabra* cultivated in three typical PDO production areas of the Calabria region and harvested during three developmental stages was performed. The influence of these factors on metabolite content was statistically determined to distinguish the patterns for cultivation area and for harvesting times. Afterwards, the in vitro antioxidant capacity of the GGL extracts and their chemical markers were evaluated, as well as their anti-aging and antioxidant properties, on a yeast model of *Saccharomyces cerevisiae* expressing human α-synuclein.

## 2. Materials and Methods

### 2.1. Chemical and Standards

Analytical grade ethanol (EtOH), MS grade formic acid (HCOOH), 6-hydroxy-2,5,7,8-acid tetramethylcroman-2-carboxylic acid (Trolox), 2,2′-azinobis (3-ethylbenzothiazoline-6-sulphonic acid (ABTS), and the reference standards of isoquercitrin and rutin were provided by Merck Chemicals (Milan, Italy). MS grade acetonitrile and water were purchased from Romil (Cambridge, UK). Ultrapure water (18 MΩ) was prepared using a Milli-Q purification system (Millipore, Bedford, TX, USA).

Pinocembrin (L29), licoflavanone (L36), glabranin (L46), dihydro-3,3′,4′-trihydroxy-5-O-prenylstilbene (L33), and dihydro-3,5,4′-trihydroxy-4,5′-diprenylstilbene (L44) were purified by RP-HPLC prepared from *G. glabra* exudate and characterized by 1D- and 2D-NMR (purity ≥ 96% from HPLC). Finally, vicenin-2 was previously isolated from natural sources [30].

### 2.2. Samples

Leaves of *G. glabra* var. *typica* (GGL) were kindly supplied by Nature Med S.r.l. (Castrovillari, CS, Italy) of the Consortium for Protected Designation of Origin “Liquirizia di Calabria PDO”. The cultivation and harvesting were conducted according to the disciplinary of the “Liquirizia of Calabria PDO”. Plant materials were collected from 3−4 year old plants in three typical PDO production areas of Calabria region: Villapiana (GGL-A), (Cosenza, Italy; coordinates (lat, long): 39.810000, 16.470000; elevation: 16 m), Castrolibero (GGL-B) (Cosenza, Italy; coordinates (lat, long): 39.322460, 16.192634; elevation: 290 m), and Scandale (GGL-C) (Crotone, Italy; coordinates (lat, long): 39.152599, 17.011761; elevation: 63 m). Leaf samples were harvested during three growing phases of 2019: Harvesting Time 1 (HT1), corresponding to the pre-flowering stage, in early May; HT 2, corresponding to the flowering stage, in mid-June; and HT 3, corresponding to the senescence stage (root collection), in late October. For each geographical area at each HT, three representative samples (about 0.5 kg each replicate) were randomly collected.

Drying of leaf materials (ventilated oven with a temperature of 50 °C) was carried out by Nature Med Seral. using standardized procedures in accordance to set rules of PDO disciplinary. Dried leaf samples were ground using a knife mill Grindomix GM 200 (Retsch, Haan, Germany) operating at intervals of brief cycles. The resulting ground materials were sieved through a test sieve with a range of 300–600 μm to obtain a sample with a homogeneous particle size distribution.

### 2.3. Ultrasound-Assisted Extraction (UAE)

Exhaustive extracts of GGL samples from each biological triplicate were prepared according to the methodology proposed by Celano et al. [17]. Briefly, 500 mg of homogenized samples was extracted in duplicate with 10 mL of aqueous ethanol (70% *v*/*v*) for 30 min at 30 °C in a thermostat-controlled ultrasound bath (Labsonic LBS2, Treviglio, Italy). The extraction process was repeated three times with fresh solvent. The extracts were pooled and diluted with distilled water to 50 mL, then filtered with 0.45 µm regenerated cellulose filters (Chromafil Xtra RC-20/13, Delchimica, Italy) for the chromatographic analysis.

For ABTS assay and biological assay, a triplicate solution of each GGL sample was freeze-dried (freeze dryer Alpha 1–2 LD, Christ, Germany) after removal of the organic solvent under vacuum at 40 °C in a rotary evaporator (Rotavapor R-200, Buchi Italia s.r.l, Cornaredo, Italy). The dried extracts were properly dissolved before assays according to procedures reported in Section 2.5 and Section 2.6.

### 2.4. Quantitative Analysis

Quantitative analysis of GGL extracts was performed using a Platin Blue UHPLC system (Knauer, Labservice Analytica, Bologna, Italy) consisting of two ultra-high-pressure pumps, an autosampler, a column temperature manager, and a diode array detector. UHPLC separation was performed using a previously developed method [17]. Briefly, the chromatographic separations were performed using a Kinetex C18 column (2.1 × 100 mm, 1.6 μm; Phenomenex, Bologna, Italy) protected by a C18 Guard Cartridge (2.1 mm I.D.) and thermostated at 25 °C. A binary gradient of H_2_O and MeCN, both containing 0.1% of formic acid, at a flow rate of 500 µL min^−1^ was employed. After each injection (5 µL), the column was washed with 98% B for 4 min and re-equilibrated (4 min). UV spectra were acquired in the range of 200–600 nm and three wavelengths were selected for the detection of target analytes based on the maximum absorbance of pure compounds (Figure S1): 220 (prenylated dihydrostilbenes), 290 (flavanones), and 360 (flavones-C-glycosides and flavonols-O-glycosides) nm.

The external standards method was employed to determine the levels of main compounds in the GGL extracts. Stock solutions of reference standards (vicenin-2, rutin, isoquercitrin, pinocembrin, licoflavanone, glabranin, dihydro-3,3′,4′-trihydroxy-5-O-prenylstilbene (L33), and dihydro-3,5,4′-trihydroxy-4,5′-diprenylstilbene (L44)) were prepared in MeOH at a concentration of 5 mg mL^−1^ and stored at −20 °C. A mixture of reference standards was prepared at a concentration of 1 mg mL^−1^, and the calibration levels were prepared by appropriate serial dilution with MeOH/H_2_O, 1:1 *v*/*v*, and analyzed in triplicate. The linearity of the calibration curves was evaluated in the concentration range of 6.25–100 µg mL^−1^ for vincenin 2, rutin, and isoquercitrin, and 12.5–200 µg mL^−1^ for the remaining reference compounds. The regression curves were tested with the analysis of variance (ANOVA), and the linear model was found to be appropriate over the tested concentration range (R^2^ values > 0.999). The samples were analyzed after appropriate dilutions to fall within the dynamic calibration range. The quantification of compounds of GGL extracts, for which no reference standards were available, was determined using the calibration curves of standards belonging to the same class of secondary metabolites (similar chromophore). The amount of the compounds was expressed as mg per g of dry leaf material (mg/g DM) ± standard deviation (n = 3 biological replicates).

### 2.5. ABTS Assay

The antioxidant activities of the exhaustive extracts and the major GGL compounds, vicenin-2, rutin, isoquercitrin, pinocembrin, licoflavanone, glabranin, dihydro-3,3′,4′-trihydroxy-5-O-prenylstilbene (L33) and dihydro-3,5,4′-trihydroxy-4,5′-diprenylstilbene (L44), were evaluated using ABTS assays adapted for use in 96-well plates, as reported in [31]. A microplate spectrophotometer reader Multiskan Go (Thermo Scientific, Milan, Italy) was employed for the assays. GGL dried extracts were dissolved in methanol at a concentration of 1 mg/mL and then diluted with 5 mM PBS (pH 7.4) to provide approximately 20–80% control absorbance. ABTS results were expressed as Trolox equivalent antioxidant capacity (TEAC) per g of extract (mmol TE/g) or per mmol of pure compound (mmol TE/mmol). In the ABTS assay, the curves of Trolox, pure compounds, and extracts were obtained by plotting concentration (mM for Trolox; standards and mg g^−1^ for leaf extracts) against the average % inhibition of radical absorbances.

### 2.6. Yeast Strains and Media

The yeast strain used to evaluate the biological activity of dried GGL extracts is BY4742 MATα his3Δ1 leu2Δ0 lys2Δ0 ura3Δ0 [pYX242-SNCA] overexpressing human α-synuclein. Cells were grown at 30 °C in minimal medium, containing 2% glucose as a carbon source and 0.67% yeast nitrogen base without amino acids, and supplemented with 50 mg/L of required amino acids and bases for which the strain was auxotrophic, with constant shaking (120 rpm). GGL extracts were resuspended in 150 µL of EtOH 100%, vortexed, incubated in the thermomixer at 25 °C for 1 h, sonicated for 20 min, dissolved in the liquid medium at a concentration of 0.2% of the yeast strain, and filtered through 0.22 µm filters, as previously reported [28,29].

### 2.7. Chronological Lifespan Experiments (CLS)

Yeast cells were grown in liquid medium until the mid-late exponential phase, centrifugated (13,000 rpm, 5 min), and then inoculated at 0.150 OD/mL (Optical Density/mL) in flasks containing medium in the absence (negative control) or presence of the extracts (0.2% concentration). Survival was assessed by propidium iodide staining (PI) at the indicated time points with a Cytoflex cytofluorimeter (Beckman Coulter, Milan, Italy) and analyzed with the Cytoflex software (version 2.6.0.105), as previously reported [28,29].

### 2.8. Analysis of Reactive Oxygen Species (ROS) Levels

Analysis of cellular superoxide was performed by DHE (dihydroethidium) staining. Cells were collected after 24 h treatment with 0.2% GGL extracts, and 0.2% were resuspended in PBS and stained with 5 μg/mL for 10 min. FACS analyses were performed with Cytoflex cytofluorimeter (Beckman Coulter, Milan, Italy) and analyzed with the Cytoflex software (version 2.6.0.105), as previously reported [28,29].

### 2.9. Statistical Analysis

The quantitative data and TEAC values were subjected to analysis of variance (ANOVA) using Statgraphic Centurion 18 software from Statgraphics Technologies (Arezzo, Italy). A two-way ANOVA (3 production areas × 3 HTs) was applied to each GGL marker, the total of metabolite classes, and TEAC values. The means were separated using the Tukey HSD test only when the F-test for factors and their interaction were significant at the *p*-value < 0.05 probability level.

With regard to the CLS assays and analysis of ROS levels, experiments were performed in duplicate. The results are expressed as mean ± SD (standard deviation). Results were compared using the two-sided Student’s *t*-test. Differences were considered statistically significant at *p* < 0.05.

## 3. Results and Discussion

### 3.1. Quantitative Profile of Glycyrrhiza glabra Leaves

Our recent study, conducted on *G. glabra* var. *typica* cultivated for the production of PDO certified Calabrian licorice, has revealed that the leaves represent a rich source of unusual bioactive compounds with potential uses in the nutraceutical, pharmaceutical, and cosmetic sectors [17].

In the current study, the quantitative profiles of *G. glabra* leaves (GGL) from different production areas of Calabria and collected at different harvest times were determined by UHPLC-UV analysis to investigate the influence of cultivation site and phenological stages on the phytochemical content of this untapped resource. GGL samples were harvested from three typical areas dedicated to the production of PDO Calabrian licorice: Villapiana (GGL-A), located near the Ionian Sea; Castrolibero (GGL-B), located in the hinterland of the Tyrrhenian coast; and Scandale (GGL-C), located further south in the hinterland of the Ionian coast. From each production area, the leaf samples were collected at three different harvesting times corresponding to three growing stages: HT1 (pre-flowering stage), HT2 (flowering stage), and HT3 (senescence stage, corresponding to the root collection) [32].

**Figure 1:**
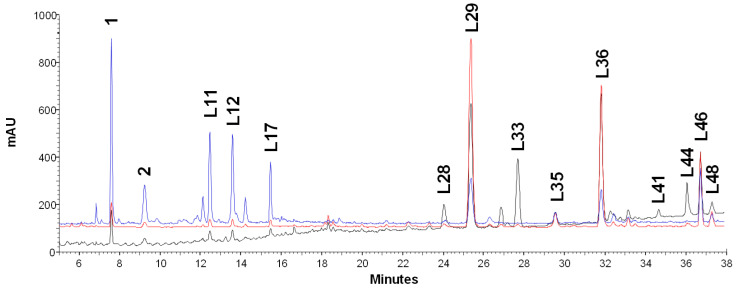
A representative UHPLC-UV profile of *G. glabra* leaves (GGL-B2): black line, 220 nm; red line, 290 nm; blue line, 360 nm.

Untargeted UHPLC-HRMS analysis [17] indicated that the qualitative profiles of investigated GGL extracts were comparable among production areas and harvesting times. The main compounds detected in UHPLC-PDA profiles (Figure 1) are as follows: the flavanones pinocembrin (L29), licoflavanone (L36), 8/6-prenylnaringenin (L35), glabranin (L46), and glabranin isomer (L48); the flavone *C*-glycosides vincenin 2 (1) and iso/shaftoside (2); the flavonol *O*-glycosides rutin (L11), isoquercitrin (L12), and astragalin (L17); and the prenylated dihydrostilbenes dihydro-3,5,3′,4′-tetrahydroxy-5′-prenylstilbene (L28), dihydro-3,3′,4′-trihydroxy-5-O-prenylstilbene (L33), dihydro-3,5,3′,4′-tetrahydroxy-4,5′-diprenylstilbene (L41), and dihydro-3,5,4′-trihydroxy-4,5′-diprenylstilbene (L44). These compounds were assumed as GGL markers and quantified in all GGL samples by UHPLC-UV analysis and external calibration method (Table S1).

**Figure 2:**
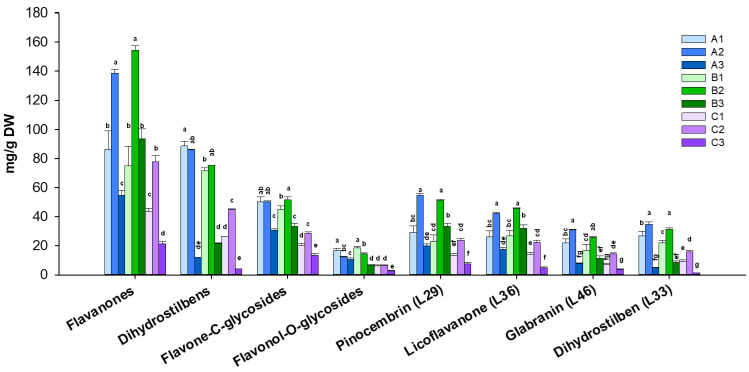
Levels of metabolite classes and the most abundant compounds from three production areas (P) and three harvest times (HT) of *G. glabra* leaves. Different letters within each metabolite class or compound indicate significant differences between extracts (*p* ≤ 0.05 by a post-hoc Tukey’s HSD test).

As reported in Table S1, the leaves of Calabrian *G. glabra* exhibited high levels of specialized metabolites, with a total content ranging from 4 to 30 g/100 g of dry leaf DM. The changes in the level of abundance of each metabolite class and the most abundant compounds (pinocembrin, licoflavanone, glabranin, and stilbene L33) are shown in Figure 2. Overall, the most abundant class of secondary metabolites was represented by flavanones, constituting 33–61% of the total content of quantified metabolites, with pinocembrin (L29) and licoflavanone (L36) being the major compounds (each accounting for 11–21% of the total content) followed by—in order of abundance—prenylated dihydrostilbenes with a content ranging from 10 to 37% of the total content (L33 and L44 as the most abundant compounds), flavone C-glycosides (17–32% of the total content), and flavonol O-glycosides (4–10% of the total content) (Table S1 and Figure 2).

The quantitative data of GGL phytochemicals revealed considerable variations in their content in relation to both the production areas (P) and harvesting times (HT) (Table S1 and Figure 2). The levels of each GGL marker and of metabolite classes were statistically evaluated according to the influence of P and HT. The two-way ANOVA analysis indicated that both P and HT statistically affected the levels of all GGL markers. Also, the interaction between the P and HT proved to be statistically significant for all the analyzed compounds and the metabolite classes (Table S1 and Table 1).

Particularly, the variation in the levels of the most abundant compound classes, flavanones and prenylated dihydrostilbenes, is predominantly attributed to the dynamic influence of development stages (HT), which shapes the overall composition of bioactive compounds according to the plant’s growth cycle. On the other hand, the contribution of P on the variance was greater for flavone *C*-glycosides and flavonol *O*-glycosides (Table 1). The effects of P and HT on the content of the different metabolite classes agree with their distinct localization in the leaf [17], suggesting a different biological role. Namely, the phytochemicals flavanones and dihydrostilbenes accumulated in the leaf exudate are likely responsible for the resistance to pathogens, herbivore attacks, and drought stress; meanwhile, glycosylated flavones and flavonols, distributed solely in the inner leaf tissue, might be involved in photoprotection, shielding plant cells from photo-oxidation [17,33].

Considering the harvesting time, the levels of GGL specialized metabolites were found to be significantly different among the three timeframes of our study (Figure S2). HT3 exhibited the lowest levels of each compound or metabolite class, regardless of the production area (Table S1 and Figure 2). These results can be easily attributed to developmental changes associated with plant growth and, in particular, with leaf senescence and the plant recycling activities of catabolism, which are necessary for nutrient remobilization from senescing leaves to sink tissues [33,34,35]. Namely, different trends in the content of the various compound classes are observed, especially for the most abundant classes. A significant variation in the flavanone levels was observed in the three HTs: both the total content and the major flavanones (pinocembrin, licoflavanone, and glabranin) more or less doubled between HT1 and HT2, and then decreased in HT 3 by 61, 40, and 73% in GGL-A, GGL-B, and GGL-C, respectively. On the contrary, the levels of prenylated dihydrostilbenes did not show relevant variations between HT1 and HT2 but underwent a drastic reduction, ranging from 71% to 91%, from HT2 to HT3, for all three production areas (Figure S2). Pinocembrin, prenylated flavanones, and dihydrostilbenes are accumulated in the sticky exudate that cover the surface of *G. glabra* leaves [17]. It has been reported that *G. glabra* exudate is most likely responsible for the resistance to biotic and abiotic stresses such as pathogens, herbivore attacks, and drought stress [36]. The well-known antimicrobial and cytotoxic activities of pinocembrin and licoflavanone further support their role in insect deterrence and defense against enemies feeding [12,13,37,38]. This is in agreement with reports on eucalypt oil glands, where pinocembrin has been identified as a main component and its biological properties, such as antibacterial, antifungal, and antifeedant activities, are clearly related to plant defense [39,40,41]. Thus, during flowering, the high levels of flavanones and prenylated dihydrostilbenes are most likely part of the defensive strategies of the licorice plant [42].

A similar variation in the flavone C-glycosides was observed, regardless of the production area. Flavonol-O-glycosides instead showed a significant content decrease in both A and B production areas from HT1 to HT2, whereas in C, their content did not significantly change during these two harvesting times (Table S1 and Figure 2). This different behavior between A/B and C production areas during the three HTs can be associated with environmental conditions. HT1 and HT2 (from late Spring to Summer) encompass seasons characterized by high UV light, especially in Southern Italy; thus, the accumulation of these antioxidant compounds is expected to increase, but our results suggest that plants located in A and B areas might experience better shading than plants growing in the C area [43].

Our data also highlight relevant differences among the different production areas. For all quantified compounds, GGL-C displayed content means that were statistically different compared to GGL-A and GGL-B; on the contrary, no statistically significant differences were observed between the A and B areas, except for the content of prenylated flavanones (Figure S2). In detail, GGL-A and GGL-B at all three studied harvest times showed the highest content of metabolites (10–29 and 15–30 g/100 g DM, respectively), whereas in GGL-C, the lowest total content of metabolites (4–16 g/100 g DM) was detected, and a statistically significant lower content of each analyzed compound was observed in these samples compared to the other two (Table S1 and Figure 2). In particular, since it is widely known that pedoclimatic conditions play a crucial role in the accumulation of secondary metabolites by influencing the uptake of water and nutrients, these results point out that the significantly lower phytochemical amounts in GGL-C samples might reflect the different, and possibly less pleasant, growth conditions experienced by *G. glabra* in the hinterland of the Ionian coast [44]. On the other hand, GGL-A and GGL-B extracts exhibited a similar content of phytochemicals, which is more than double compared to GGL-C. Generally, no marked differences among the different metabolite classes were observed.

Overall, the quantitative profile of GGL indicates that this untapped resource can provide a bioactive-rich matrix if harvested in the A and B areas and at the first two harvesting times.

### 3.2. Antioxidant Activity In Vitro and In Vivo of Glycyrrhiza glabra Extracts

**Figure 3:**
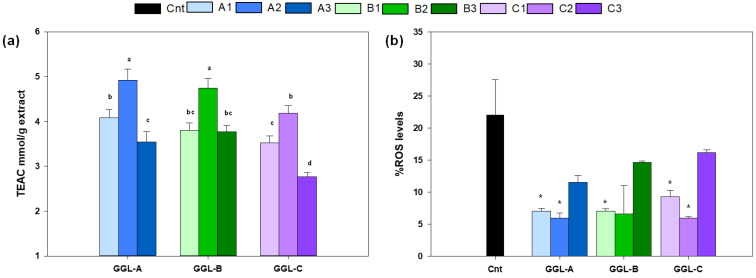
Antioxidant capacity of *G. glabra* leaf extracts from three production areas and three harvest times, measured as (**a**) TEAC equivalents by ABTS assay and (**b**) ROS content (assayed by DHE staining) of yeast cells BY4742 bearing pYX242-SNCA plasmid grown in minimal medium containing 2% glucose for 24 h in the absence (cnt) or presence of 0.2% *G. glabra* leaf extracts. Different letters in (**a**) indicate significant differences between extracts (*p* ≤ 0.05 by Tukey’s HSD test). * *p* < 0.05 relative to control cells. Data of (**b**) represent mean and standard deviation of at least two independent experiments.

In order to evaluate how the differences recorded in GGL phytochemical profiles reflect biological properties, the antioxidant capacity of GGL extracts was evaluated spectrophotometrically by ABTS in vitro assay (Figure 3a). Furthermore, to ascertain which specialized metabolites mostly contribute to the antioxidant activity, distinctive GGL compounds were assayed for their antioxidant potential (Table 2).

Also, for in vitro antioxidant capacity, two-way ANOVA analysis revealed a statistically significant variation across the production area (P) and according to the harvesting time, as well as the interaction P × HT (Table 1). Antioxidant activity of GGL extracts (Figure 3a) changed according to the quantitative profiles. Regarding the harvest time, GGL samples collected in HT 2 showed significantly higher antioxidant activities than HT1 and HT3 (Figure S2), consistent with the metabolite enrichment characteristic of this growth stage. Conversely, no statistically significant differences were observed between the A and B areas (Figure S2). These data further support the best harvest conditions identified by the quantitative analysis of GGL markers, suggesting that these compounds are most likely responsible for antioxidant activity. Indeed, as shown in Table 2, the tested compounds showed higher antioxidant powers than Trolox, with the following activity order: licoflavanone > isoquercitrin > rutin > stilbene L33 > glabranin > pinocembrin > stilbene L44 > vincenin 2. Interestingly, the most abundant compounds (licoflavanone, stilbene L33, glabranin, and pinocembrin) showed superior or comparable antioxidant activity to the flavonols rutin and isoquercetin, the content of which was distinctly lower in the GGL extracts. Thus, the antioxidant capacity of GGL might be attributed to the high content of prenylated flavanones and dihydrostilbenes. These results are in agreement with what has been reported in previous studies [15,45,46] on the bioactivity of *G. glabra* leaf extracts.

Oxidative stress, resulting from the excessive intracellular accumulation of reactive oxygen species (ROS), reactive nitrogen species (RNS), and other free radical species, contributes to the onset and progression of various diseases, among which is Parkinson’s disease (PD) [47]. Based on results of the in vitro antioxidant capacity of *G. glabra* leaves and its main specialized metabolites and in order to estimate the ability of GGL extracts to exert an antioxidant response at the cellular level, not just their radical scavenging capacity, the antioxidant effect of the GGL extracts was evaluated on a yeast model of *Saccharomyces cerevisiae* expressing human α-synuclein. As shown in Figure 3b, the treatment with GGL extracts remarkably reduced the intracellular ROS level in exponentially growing yeast cells, with significant antioxidant effects for the GGL extracts with the highest phytochemical contents (harvesting times 1 and 2).

### 3.3. Anti-Aging Effects of Glycyrrhiza glabra Extracts in Yeast Cells Expressing Human α-Synuclein

**Figure 4:**
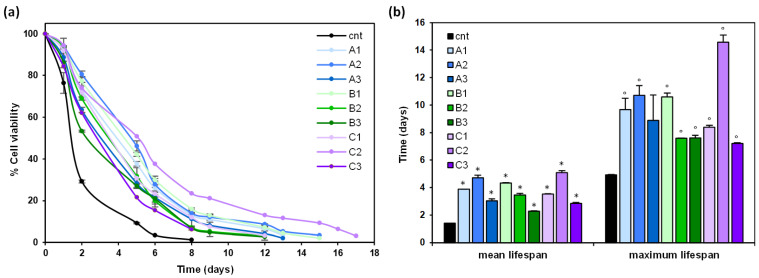
Effect of *G. glabra* leaf extracts on α-synuclein toxicity: (**a**) chronological lifespan (CLS) of yeast cells BY4742 bearing pYX242-SNCA plasmid grown in minimal medium containing 2% glucose in the absence (cnt) or presence of 0.2% GGL extracts; (**b**) mean and maximum lifespan of CLS of yeast cells as in (**a**). * *p* < 0.05 relative to mean lifespan in control cells; ° *p* < 0.05 relative to maximum lifespan in control cells.

α-Synuclein is a presynaptic protein associated with the pathophysiology of synucleinopathies, including Parkinson’s disease [23,48], and budding yeast has been extensively employed in models of synucleinopathies [25,26]. Thus, the effect of GGL extracts on the longevity of yeast cells over-expressing the human α-synuclein was evaluated. In order to evaluate whether GGL extracts could exert anti-aging effects, they were added to exponentially growing yeast cells expressing human α-synuclein at a concentration of 0.2% in a synthetic minimal medium. Results show that all the extracts prolonged the chronological lifespan (CLS) of yeast cells (Figure 4a), with a significant marked increase in both mean and maximal lifespan. In fact, mean lifespan resulted 2.5 times longer while maximal lifespan increased up to 3 times relative to the control (Figure 4b).

Overall, the data presented indicate that *G. glabra* extracts delay senescence by reducing intracellular ROS level and α-synuclein toxicity.

## 4. Conclusions

In this study, we explored the influence of different harvest times and cultivation areas on the phytochemical content of *G. glabra* leaves, showing a direct influence of these factors on specialized metabolites production. According to the results, different harvest periods have a strong impact on the concentration of bioactive compounds, especially on flavanones and dehydrostilbenes, thus also influencing GGL extracts’ bioactivities.

Regarding the antioxidant and antiaging power of GGL extracts, our results clearly indicate that harvesting time is essential for obtaining desired bioactive chemical constituents. Aging and age-related neurodegeneration are among the major challenges in modern medicine because of the progressive increase in the number of elderly in the world population. Nutrition, which has evident long-term consequences for health, is an important way to prevent diseases and achieve healthy aging. Our study successfully demonstrates the beneficial effect of GGL extracts on senescence related to α-synuclein toxicity along with their notable antioxidant properties, supporting the role of GGL extracts as a functional food in age-related disorders.

Overall, this study provides important theoretical foundations and technical support for selecting the best cultivation area in order to optimize *G. glabra* cultivation in the Calabria region, also offering the possibility to envisage the most favorable harvest time for valorization of *G. glabra* leaf resources according to the principles of sustainable development in agricultural processes.

## Figures and Tables

**Table 1:** F ratios of production area (P) and harvest time (HT) and their interaction of specialized metabolites content in *G. glabra* leaves ^a^.

Factor	Flavanones	Dihydrostilbenes	Flavones	Flavonols	L29	L36	L46	L33	TEAC
P (2) ^b^	183 *	132 *	417 *	294 *	210 *	211 *	101 *	216 *	40 *
HT (2) ^b^	239 *	318 *	202 *	182 *	250 *	171 *	168 *	531 *	114 *
P × HT (4) ^b^	15 *	21 *	9 *	27 *	26 *	13 *	11 *	25 *	3.4 *

^a^ Values are given as F-ratio; * denote significant at *p* ≤ 0.001 of Fisher; ^b^ degrees of freedom in brackets.

**Table 2:** Antioxidant activity of GGL main compounds by ABTS assay ^a^.

Compound	TEAC (mmol TE/mmol ± SD)
Vincenin 2	1.12 ± 0.01
Rutin	6.00 ± 0.04
Isoquercitrin	6.45 ± 0.03
Pinocembrin	3.21 ± 0.01
Licoflavanone	9.55 ± 0.04
Glabranin	4.08 ± 0.03
Prenylstilbene (L33)	5.09 ± 0.01
Prenylstilbene (L44)	2.50 ± 0.01

^a^ Values are means of three replicates.

## Data Availability

Data are contained within the article. Further inquiries can be directed to the corresponding author.

## Supplementary Materials

The following supporting information can be downloaded at: https://www.mdpi.com/article/10.3390/antiox13010093/s1, Table S1: Level of the main *G. glabra* leaves according to the production area (P) and harvesting time (HT) on metabolite content of *G. glabra* leaves; Figure S1: UV spectra of main GGL compounds; Figure S2: Means plot of metabolite contents and TEAC of *G. glabra* leaves according to the production area (P) and harvesting time (HT).
