# Supplementary material for: Exploring Influence of Production Area and Harvest Time on Specialized Metabolite Content of Glycyrrhiza glabra Leaves and Evaluation of Antioxidant and Anti-Aging Properties
Source: Antioxidants (Basel). 2024 Jan 12;13(1):93. doi: 10.3390/antiox13010093 (PMC10812728; doi:10.3390/antiox13010093)
Supplement: Supplementary file 1 [file antioxidants-13-00093-s001.zip › antioxidants-2805847-supplementary.pdf]

Supplementary material

of

Exploring Influence of Production Area and Harvest Time on Specialized Metabolite Content of *Glycyrrhiza glabra* Leaves and Evaluation of Antioxidant and Anti-Ageing Properties

Teresa Docimo [1], Rita Celano [2,3,*], Alessia Lambiase [3,4], Rosa Di Sanzo [5], Simona Serio [2,6], Valentina Santoro [2,3], Paola Coccetti [3,4], Mariateresa Russo [5], Luca Rastrelli [2,3] and Anna Lisa Piccinelli [2,3]

1 Institute of Bioscience and BioResources, National Research Council, 80055 Portici, Italy; teresa.docimo@ibbr.cnr.it
2 Department of Pharmacy, University of Salerno, Via Giovanni Paolo II 132, 84084 Fisciano, Italy; sserio@unisa.it (S.S.); vsantoro@unisa.it (V.S.); rastrelli@unisa.it (L.R.); apiccinelli@unisa.it (A.L.P.)
3 National Biodiversity Future Center (NBFC), 90133 Palermo, Italy; a.lambiase1@campus.unimib.it (A.L.); paola.coccetti@unimib.it (P.C.)
4 Department of Biotechnology and Biosciences, University of Milano-Bicocca, 20126 Milano, Italy
5 Department of Agriculture Science, Food Chemistry, Safety and Sensoromic Laboratory (FoCuSS Lab), University of Reggio Calabria, Via Salita Melissari, 89124 Reggio Calabria, Italy; rosa.disanzo@unirc.it (R.D.S.); mariateresa.russo@unirc.it (M.R.)
6 PhD Program in Drug Discovery and Development, University of Salerno, Via Giovanni Paolo II 132, 84084 Fisciano, Italy
* Correspondence: rcelano@unisa.it

Table S1. Level of the main *G. glabra* leaves according to the production area (P) and harvesting time (HT) on metabolite content of *G. glabra* leaves.

	GGL compound [1,2] (mg/g of dry leaf)													
	**1**	**2** [3]	**L11**	**L12**	**L17** [4]	**L29**	**L36**	**L35** [5]	**L46**	**L48** [6]	**L33**	**L44**	**L28** [7]	**L41** [8]
**GGL-A*1**	34.7 ^{a}	15.4 ^{a}	6.7 ^{a}	4.1 ^{ab}	6.0 ^{b}	29.2 ^{bc}	26.0 ^{bc}	4.5 ^{b}	21.8 ^{bc}	4.9 ^{de}	26.7 ^{b}	33.2 ^{a}	11.0 ^{b}	17.6 ^{a}
**GGL-A*2**	35.3 ^{a}	14.9 ^{a}	4.5 ^{b}	2.8 ^{c}	4.9 ^{bc}	54.5 ^{a}	42.1 ^{a}	3.5 ^{c}	31.0 ^{a}	7.7 ^{c}	34.8 ^{a}	29.6 ^{a}	12.8 ^{a}	8.8 ^{bc}
**GGL-A*3**	22.6 ^{c}	8.1 ^{bc}	3.4 ^{c}	2.8 ^{c}	4.5 ^{c}	19.9 ^{de}	17.6 ^{de}	2.4 ^{d}	7.8 ^{fg}	6.8 ^{cd}	4.8 ^{fg}	3.9 ^{cd}	2.8 ^{e}	0.5 ^{ef}
**GGL-B*1**	30.0 ^{b}	15.1 ^{a}	6.8 ^{a}	4.2 ^{a}	7.5 ^{a}	23.2 ^{cd}	26.7 ^{bc}	4.8 ^{ab}	16.9 ^{cd}	3.5 ^{e}	22.1 ^{c}	27.1 ^{ab}	10.5 ^{b}	11.8 ^{b}
**GGL-B*2**	36.3 ^{a}	15.2 ^{a}	5.3 ^{b}	3.6 ^{b}	5.6 ^{bc}	51.3 ^{a}	45.6 ^{a}	5.4 ^{a}	25.6 ^{b}	26.6 ^{a}	31.4 ^{a}	26.2 ^{ab}	11.7 ^{ab}	5.9 ^{cd}
**GGL-B*3**	23.2 ^{c}	10.1 ^{b}	2.2 ^{d}	2.1 ^{d}	2.4 ^{d}	33.3 ^{b}	32.0 ^{b}	3.2 ^{cd}	11.2 ^{ef}	13.7 ^{b}	8.6 ^{ef}	6.5 ^{cd}	4.2 ^{cd}	2.3 ^{def}
**GGL-C*1**	13.3 ^{d}	7.1 ^{c}	2.2 ^{d}	1.5 ^{e}	2.6 ^{d}	13.4 ^{ef}	14.0 ^{e}	1.5 ^{e}	7.3 ^{fg}	7.3 ^{c}	9.4 ^{e}	11.0 ^{c}	3.2 ^{de}	2.8 ^{def}
**GGL-C*2**	20.5 ^{c}	8.2 ^{bc}	1.9 ^{d}	1.7 ^{de}	3.1 ^{d}	23.8 ^{cd}	22.3 ^{cd}	2.9 ^{cd}	14.4 ^{de}	14.5 ^{b}	15.9 ^{d}	19.9 ^{b}	5.0 ^{c}	4.3 ^{de}
**GGL-C*3**	9.6 ^{e}	3.9 ^{d}	0.9 ^{e}	0.8 ^{f}	1.1 ^{e}	7.6 ^{f}	4.8 ^{f}	1.2 ^{e}	3.8 ^{g}	4.0 ^{e}	1.3 ^{g}	1.4 ^{d}	1.1 ^{f}	0.3 ^{f}
	**Analysis of Variance (F-ratio, * p < 0.01)**													
**P (2)** [9]	493 *	212 *	285 *	348 *	146 *	210 *	211 *	155 *	101 *	286 *	216 *	41 *	493 *	56 *
**HT (2)** [9]	223 *	127 *	207 *	136 *	100 *	250 *	171 *	69 *	168 *	521 *	531 *	158 *	634 *	120 *
**P x HT (4)** [9]	10 *	5 *	25 *	26 *	22 *	26 *	13 *	14 *	11 *	179 *	25 *	10 *	47 *	25 *

[1] vincenin 2 (1), iso/shaftoside (2), rutin (L11), isoquercitrin (L12), astragalin (L17), pinocembrin (L29), licoflavanone (L36), 8/6-prenylnaringenin (L35), glabranin (L46), glabranin isomer (L48), dihydro-3,3',4'-trihydroxy-5-O-prenylstilbene (L33), dihydro-3,5,4′-trihydroxy-4,5′-diprenylstilbene (L44), dihydro-3,5,3′,4′-tetrahydroxy-5′-prenylstilbene (L28), dihydro-3,5,3′,4′-tetrahydroxy-4,5′-diprenylstilbene (L41); [2] for a given compound, different superscript letters in the same column indicate significantly different values ($P \leq 0.05$ by a post-hoc Tukey's HSD test); [3] levels *of 2* are expressed as *vincenin* equivalent; [4] levels *of L17* are expressed as *vincenin* equivalent; [5] L35 levels are expressed as *licoflavanone* equivalent; [6] L48 levels are expressed as *glabranin* equivalent; [7] L28 levels are expressed as *stilbene L33* equivalent; [8] L*41* levels are expressed as *stilbene L44;* [9] degrees of freedom in brackets.

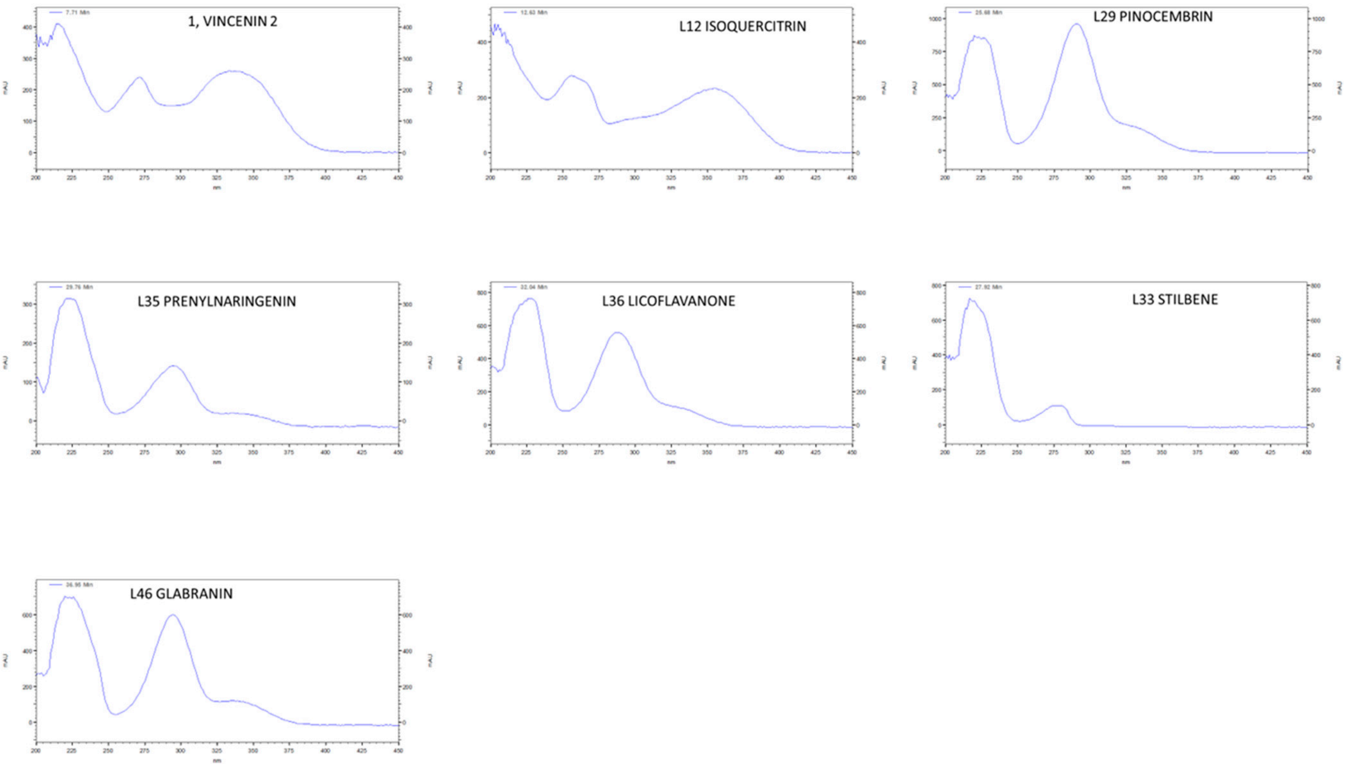

Figure S1. UV spectra of GGL markers.

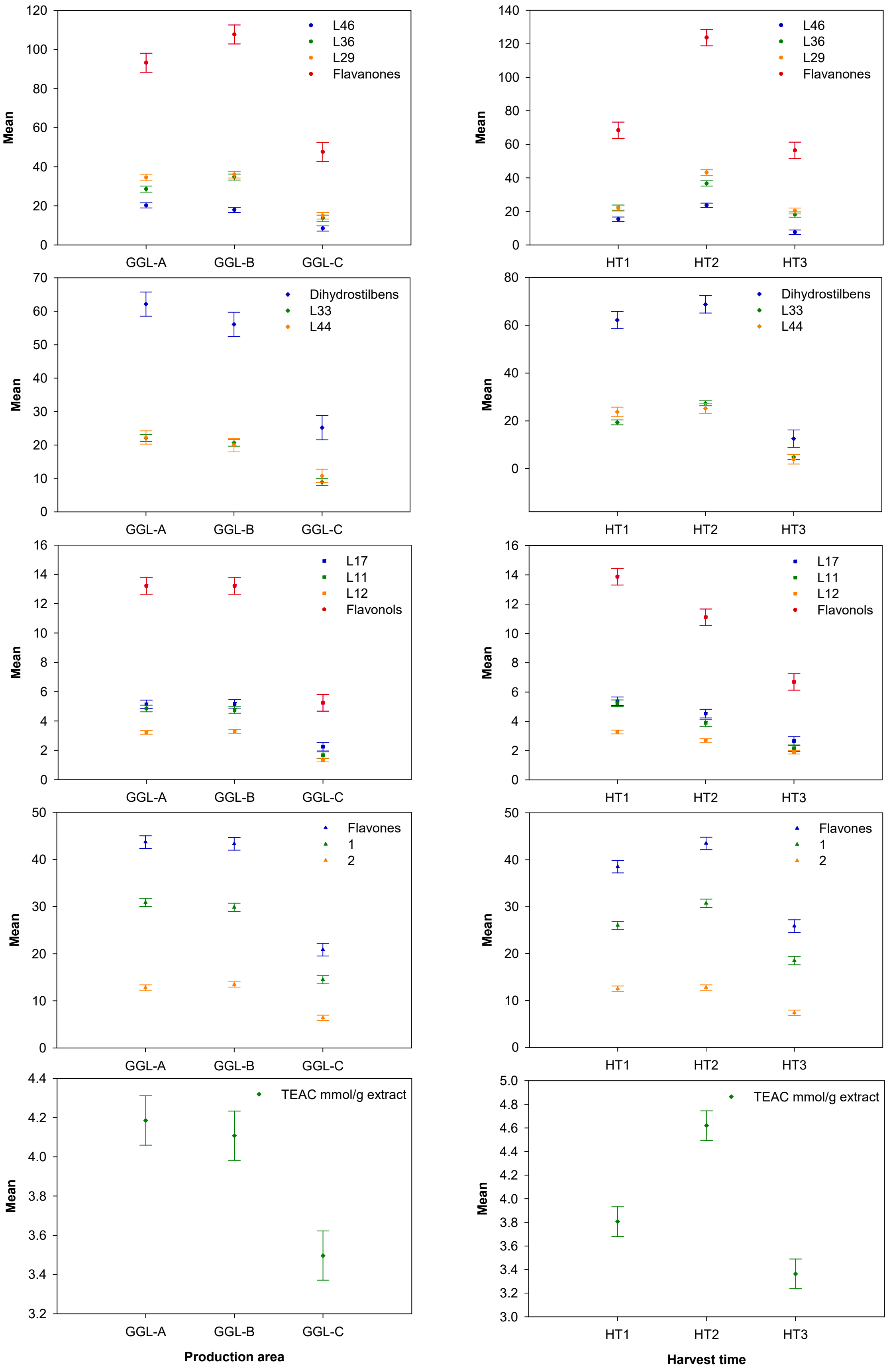

[a] Bars display the confidence intervals for the level means using the mean squared error from the ANOVA. A pair of intervals that do not overlap indicates a statistically significant difference between the means at the selected confidence level ($p < 0.05$).

Figure S2. Means plot of metabolite contents and TEAC of *G. glabra* leaves according to the production area (P) and harvesting time (HT). [a]
